# Prognostic Role of the MicroRNA-200 Family in Various Carcinomas: A Systematic Review and Meta-Analysis

**DOI:** 10.1155/2017/1928021

**Published:** 2017-02-22

**Authors:** Jung Soo Lee, Young-Ho Ahn, Hye Sung Won, Der Sheng Sun, Yeo Hyung Kim, Yoon Ho Ko

**Affiliations:** ^1^Department of Rehabilitation Medicine, College of Medicine, The Catholic University of Korea, Seoul, Republic of Korea; ^2^Department of Molecular Medicine and Tissue Injury Defense Research Center, Ewha Womans University School of Medicine, Seoul, Republic of Korea; ^3^Division of Oncology, Department of Internal Medicine, College of Medicine, The Catholic University of Korea, Seoul, Republic of Korea; ^4^Cancer Research Institute, College of Medicine, The Catholic University of Korea, Seoul, Republic of Korea

## Abstract

*Background/Aims.* The miRNA-200 (miR-200) family may act as key inhibitors of epithelial-to-mesenchymal transition. However, the potential prognostic value of miR-200s in various human malignancies remains controversial. This meta-analysis analyzed the associations between miR-200 levels and survival outcomes in a variety of tumors.* Methods.* Eligible published studies were identified by searching the Embase, PubMed, CINAHL, and Google scholar databases. Patient clinical data were pooled, and pooled hazard ratios (HRs) with 95% confidence intervals (95% CI) were used to calculate the strength of this association.* Results.* The pooled HRs suggested that high tissue expression of miR-200 family members was associated with better survival (overall survival [OS]: HR = 0.70, 95% CI 0.54–0.91; progression-free survival [PFS]: HR = 0.63, 95% CI 0.52–0.76) in thirty-four eligible articles. In contrast, higher expression of circulating miR-200 members was significantly associated with poor clinical outcome (OS, HR = 1.68, 95% CI 1.15–2.46; PFS, HR = 2.62, 95% CI 1.68–4.07).* Conclusion.* The results from this meta-analysis suggest that miR-200 family members are potential prognostic biomarkers in patients with various carcinomas. To apply these findings in the clinic, large prospective studies are needed to validate the prognostic values of miR-200s in individual cancer types.

## 1. Introduction

MicroRNAs (miRNAs) are a class of small (19–22 nucleotides), endogenous, noncoding, highly conserved, and single-stranded RNAs. miRNAs negatively regulate numerous genes by forming base-pairs with target mRNAs, thereby facilitating translational silencing or mRNA degradation of targeted genes [1]. The miRNA binding sites, complementary sequences within the 3′-untranslated regions of target genes, are critical for the regulatory effects of miRNAs on gene expression [1]. MiRNAs are implicated in regulating many fundamental and biological processes such as cellular differentiation, proliferation, metabolism, cell-cycle control, and apoptosis [2]. MiRNAs frequently reside in fragile sites and genomic regions involved in various cancers, suggesting that they play a potentially critical and complex role in cancer [3]. Unique miRNA expression profiles have been observed in various cancer types. In addition, miRNAs may act as tumor suppressors or oncogenes in cancer and can influence the response to treatment [4].

The miR-200 family includes five members (miR-200a, miR-200b, miR-200c, miR-141, and miR-429) and can be divided into two clusters based on chromosomal location. The miR-200b/a/429 cluster is comprised of miR-200a, miR-200b, and miR-429 and is located on chromosome 1p36. The miR-200c/141 cluster is comprised of miR-200c and miR-141 and is located on chromosome 12p13 [5]. MiR-200b, miR-200c, and miR-429 have the same seed region (nucleotides 2–7), and miR-200a and miR-141 share a seed region with a difference in only the fourth nucleotide (U to C) among these regions [6]. The miR-200 family was first reported to play a role in olfactory neurogenesis [7]. A number of studies showed that miR-200 family members are aberrantly expressed in multiple human malignancies, suggesting that these miRNAs play a role in tumor pathogenesis during all stages of carcinogenesis. The miR-200 family acts as key inhibitors of epithelial-to-mesenchymal transition (EMT) by directly targeting transcriptional repressors of E-cadherin, ZEB1, and ZEB2 [5]. MiR-200 family members are also likely downregulated during tumor progression. In addition, these miRNAs suppress cell proliferation by inhibiting self-renewal and differentiation of cancer stem cells and modulating cell division and apoptosis. These finding suggest that the miR-200 family members function as tumor suppressor genes. The tumor-suppressive roles of the miR-200 family have also been reported in gastric [8], breast [9], endometrial, [10] pancreatic cancers [11, 12], hepatocellular carcinoma [13], gliomas [14], and lung cancer [15, 16].

EMT, thought to play a fundamental role during tumorigenesis, is associated with poor histological differentiation, local invasiveness, and distant metastasis in various cancers. Thus, expression of miR-200 family members could influence the cancer phenotype and prognosis of cancer patients [5]. However, due to small sample sizes and different detection methods used in previous studies, the prognostic role of miR-200 has not been clearly elucidated. The discovery of molecular prognostic factors could contribute to classifying patients by prognosis and identifying high-risk cases requiring aggressive approaches. Meta-analyses offer increasing statistical power and resolve any inconsistencies or discrepancies among different studies. Therefore, we performed a literature-based meta-analysis of eligible studies to obtain evidence-based results on the prognostic role of miR-200 family members in various types of malignancies.

## 2. Materials and Methods

### 2.1. Search Strategy and Selection Criteria

We searched the CINAHL, Embase, and Google scholar using the defined keywords and PubMed using medical subject headings (MeSH) vocabulary to identify relevant articles up to December 2015. The articles were searched using the following keywords and MeSH vocabulary (Supplementary Table 1 in Supplementary Material available online at https://doi.org/10.1155/2017/1928021): miR-141, miR-200, or miR-429 combined with prognostic, prognosis, survival, tumor, cancer, neoplasm, or carcinoma. We also conducted a manual search. Articles meeting the following criteria were included: (1) human patient versus animal study on any type of malignant cancer or neoplasm and (2) assessment data on patient survival (overall survival [OS] and progression-free survival [PFS]) and the miR-200 family with multivariate hazard ratios (HRs) included. Exclusion was based on the following criteria: (1) review articles, letters, or abstracts, (2) no appropriate data, and (3) non-English or unpublished articles. The statistical data were reviewed before inclusion in the final selection, and the study data were extracted based on a predefined standardized form.

### 2.2. Data Extraction, Quality Assessment, and Statistical Methods

For the meta-analysis, the effect size was evaluated using multivariate HRs with 95% confidence intervals (95% CIs) for OS or PFS according to high miRNA expression. OS was measured from the time at which the baseline blood or tissue sample was obtained to the date of death from any cause or the date of last follow-up. PFS was measured as the time between the baseline blood and tissue sampling for miRNA analysis and documentation of the first tumor progression, based on clinical and radiological findings or death (events).

Two reviewers systematically evaluated the assessment of all selected studies according to the Newcastle-Ottowa Scale for the quality assessment of articles [42]. The study information was collected using a predefined form. The meta-analysis statistics were obtained using Revman (version 5.3.5). Heterogeneity of the combined HRs was assessed using Cochran's *Q* test and Higgins *I*-squared statistic. A *P* value less than 0.1 was considered statistically significant. A random effect model (DerSimonian and Laird method) was applied if heterogeneity was observed among studies (*P* < 0.1), while the fixed-effects model was used if no heterogeneity was observed (*P* > 0.1). Publication bias was evaluated using the funnel plot with Egger's bias indicator test [43].

## 3. Results

### 3.1. Literature Selection

**Figure 1:**
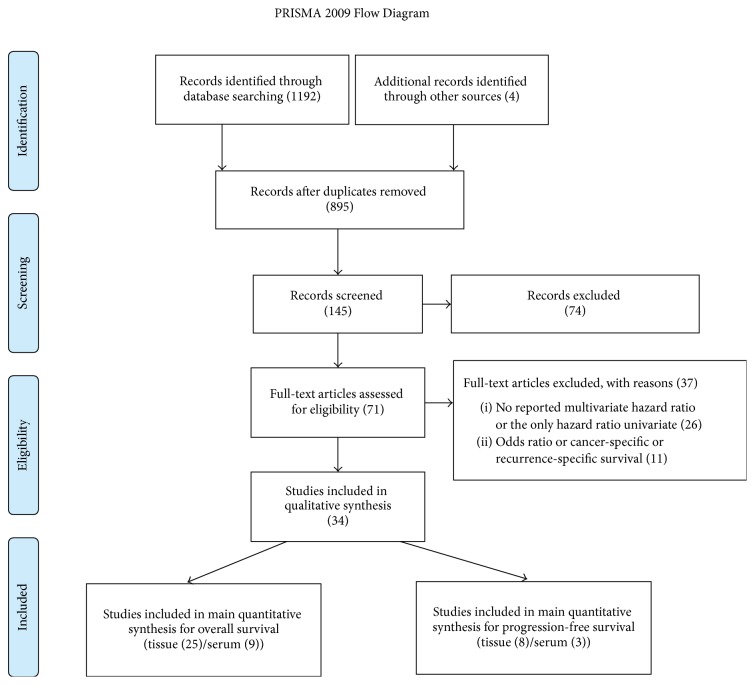
Flow chart of the selection process of the eligible articles.

After removal of duplicates, 895 studies were identified from the searches in the PubMed, CINAHL, Embase, and Google scholar databases. 750 studies were excluded using these criteria; unpublished, non-English, letters or abstracts, withdrawn articles, review articles, nonhuman studies, or irrelevant to the current analysis. Of the remaining 145 studies, 74 were excluded because they did not have the survival data associated with miR-200 family. Of the remaining 71 studies, 26 did not include the data of hazard ratio associated with OS or PFS data, and 11 included odds ratio or univariate Cox regression HRs for survival data. Finally, 34 eligible studies were selected for the final analysis. A flow chart depicting the article selection process is shown in Figure 1.

### 3.2. Literature Characteristics

The main features of the 34 enrolled studies are systematically summarized in Tables 1 and 2. Briefly, these studies were published between 2011 and 2015, and the study sample sizes ranged from 30 to 373 (median 105.5) patients. A total of 4497 patient samples were included. Patient OS data were reported in 33 studies, PFS data in 11, and both OS and PFS data in 10. All studies were nonrandomized and retrospective except for one prospective study. The malignant neoplasms assessed in these studies included brain, breast, colorectal, endometrial, esophageal, gastric, hepatocellular, non-small-cell lung, ovarian, pancreatic, and prostate cancers. Nineteen cohorts staged with I–IV cancers were included. Quantitative real-time PCR was performed in 22 studies, in situ hybridization in 2 studies, and two separate techniques in 2 studies to assess miR-200 family expression. Tissue (in 26 studies), serum (in 9 studies), and both tissue and serum samples (in 1 study) were used to determine miR-200 expression.

### 3.3. Quality Assessment and Meta-Analysis

We systematically assessed the quality of all non-randomized studies included in the meta-analysis based on the Newcastle-Ottawa Scale criteria. The following aspects of each study were evaluated based on the (1) selection of the study groups, (2) comparability of the groups, and (3) ascertainment of either the exposure or outcome of interest. These criteria were assessed on a star scoring system, with higher scores given to higher-quality studies. The quality assessment is summarized in Tables 1 and 2.

### 3.4. Overall Effects of miR-200 Expression in Cancer Tissues on OS and PFS

**Figure 2:**
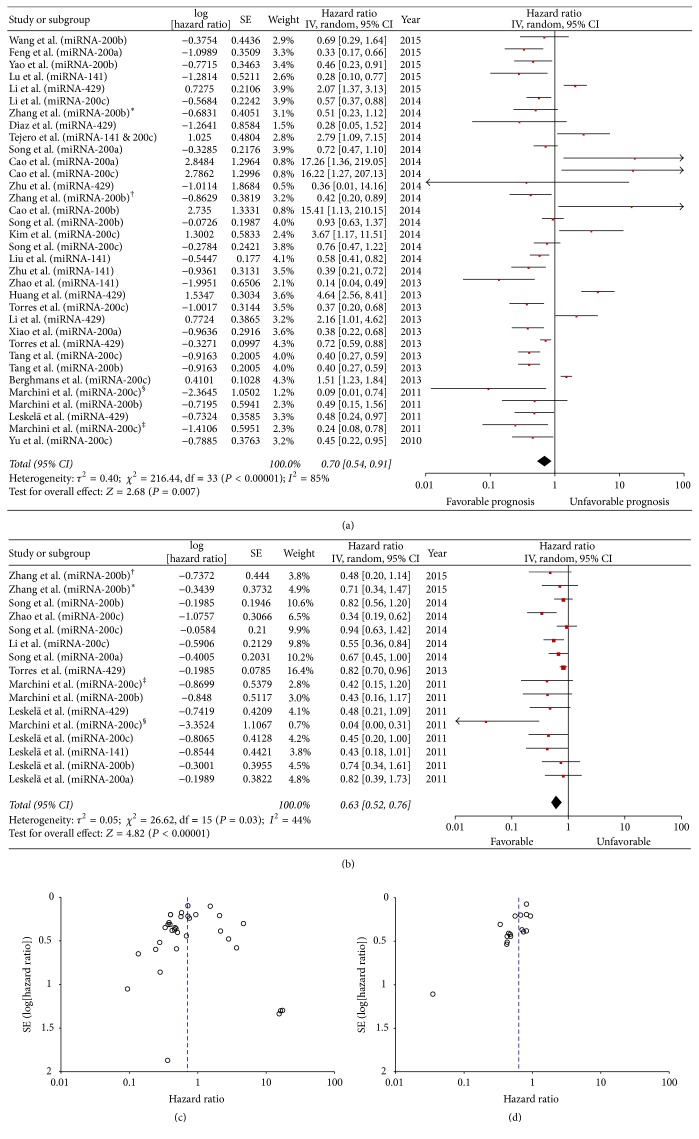
Forest plot of hazard ratios for the prediction of overall (a) and progression-free survival (b) by high-expressing miR-200 family members in tissue samples. Funnel plot showing publication bias of the overall (c) and progression-free survival (d) prediction by high-expressing miR-200 family members in tissue samples. ^*∗*^Sample from grade IV astrocytoma. ^†^Sample from grade III astrocytoma.^‡, §^Samples from different tissue collection.

Because a growing body of evidence suggests that miRNA function differs between cancer tissue and blood [44, 45], the prognostic role of miR-200 family members in both tumor tissue and serum was evaluated. Twenty-five studies on miR-200 expression in tissue samples were evaluated for OS analysis (Figure 2(a)) using a random-effects model due to high heterogeneity (OS, *P* < 0.00001, *I*^2^ = 85%). Pooled HRs and 95% CIs were calculated. The pooled results showed that high miR-200 expression was a favorable prognostic factor in patients with various types of cancer (pooled HR = 0.70, 95% CI 0.54–0.91). In addition, the PFS analysis of seven studies revealed a protective role for increased miR-200 tissue expression (pooled HR = 0.63, 95% CI 0.52–0.76), as determined using a random-effects model (*P* = 0.03, *I*^2^ = 44%; Figure 2(b)).

### 3.5. Overall Effects of Circulating miR-200 Expression on OS and PFS

**Figure 3:**
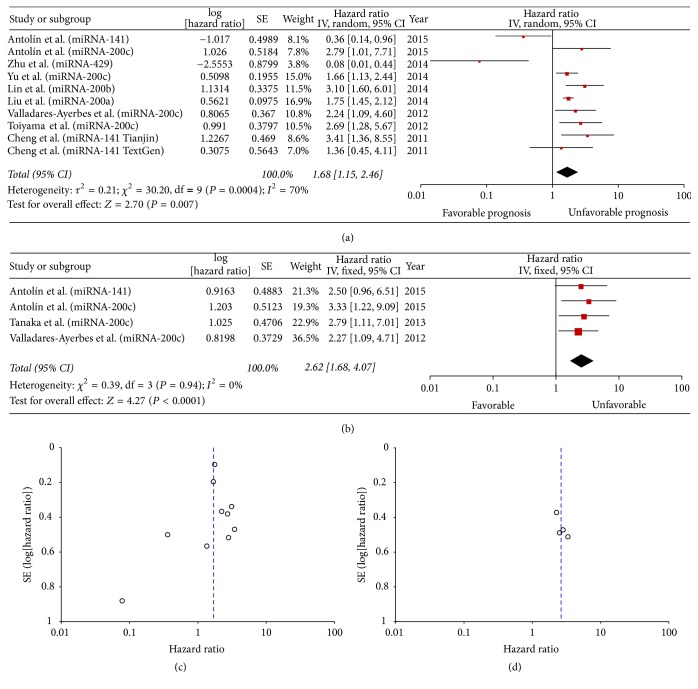
Forest plot of hazard ratios for the prediction of overall (a) and progression-free survival (b) by high-expressing miR-200 family members in serum samples. Funnel plot showing publication bias of the overall (c) and progression-free survival (d) prediction by high-expressing miR-200 family members in serum samples.

The prognostic role of circulating miR-200 family members on OS was evaluated in eight studies, and heterogeneity was apparent among studies (*P* = 0.0004, *I*^2^ = 70%). We found that higher expression of circulating miR-200 significantly predicted poor OS (pooled HR = 1.68, 95% CI 1.15–2.46; Figure 3(a)). PFS analysis of three studies (Figure 3(b)) demonstrated a significant association between circulating miR-200 levels and PFS (pooled HR = 2.62, 95% CI 1.68–4.07).

### 3.6. Subgroup Analyses of OS and PFS

To evaluate intrastudy inconsistencies and heterogeneity, the studies were stratified by the variables shown in Table 1. The heterogeneity decreased in meta-analyses of OS and PFS when the studies were stratified by the primary tumor site and individual miRNA. Pooled analyses of the brain tumor and pancreatic cancer subgroups indicated that tissue miR-200 family expression was positively correlated with OS (pooled HR = 0.51, 95% CI 0.32–0.82 in brain tumor subgroup; pooled HR = 0.35, 95% CI 0.21–0.60 in pancreatic cancer subgroup), with low heterogeneity among the studies analyzed (*P* = 0.71, *I*^2^ = 0% in brain tumor subgroup; *P* = 0.26, *I*^2^ = 26% in pancreatic cancer subgroup; Supplementary Figure  1A). In the stratified analyses of PFS, increased tissue miR-200 expression was significantly associated with increased PFS in the ovarian cancer subgroup (pooled HR = 0.50, 95% CI 0.35–0.72) with low heterogeneity (*P* = 0.26, *I*^2^ = 21%; Supplementary Figure  1B). In contrast, a pooled analysis of the colorectal cancer subgroup showed that serum miR-200 expression was negatively correlated with OS (pooled HR = 2.50, 95% CI 1.50–4.18) with low heterogeneity (*P* = 0.44, *I*^2^ = 0%; Supplementary Figure  2A). In the breast cancer subgroup, circulating miR-200 expression showed a significantly negative correlation with PFS (pooled HR = 2.87, 95% CI 1.43–5.73) with low heterogeneity (*P* = 0.69, *I*^2^ = 0%, Supplementary Figure  2B).

Among the subgroup analyses stratified by individual miRNAs, a pooled analysis of the miR-141 subgroup indicated that increased tissue expression was significantly correlated with enhanced OS (pooled HR = 0.38, 95% CI 0.23–0.64), which was determined using a random-effects model given the moderate heterogeneity among the studies (*P* = 0.09, *I*^2^ = 53%; Supplementary Figure  3A). In addition, the high miR-200b subgroup showed a longer PFS than that of the low miR-200b subgroup (pooled HR = 0.71, 95% CI 0.54–0.94), which was determined using a fixed-effects model given the low heterogeneity among the studies (*P* = 0.68, *I*^2^ = 0%; Supplementary Figure  3B). In contrast, the analysis stratified by circulating miRNA levels showed that circulating miR-200c expression was negatively correlated with OS (pooled HR = 1.97, 95% CI 1.47–2.65; Supplementary Figure  4A) and PFS (pooled HR = 2.65, 95% CI 1.61–4.35) which was determined using a fixed-effects model given the low heterogeneity among the studies (*P* = 0.83, *I*^2^ = 0%; Supplementary Figure  4B).

## 4. Discussion

MiRNAs have numerous advantages over mRNAs for predicating clinical outcomes in cancer patients, because miRNAs are posttranscriptional regulators of multiple target genes and are involved in various cellular pathways [1]. Thus, miRNAs potentially regulate complex biological processes and biomarkers involved in cancer prognosis [4]. Although the miR-200 family is a determinant of epithelial cell phenotypes, its prognostic role has not yet been elucidated. In addition, increasing evidence suggests that miRNAs have different roles in tumor tissues and blood [44, 45], and thus the prognostic roles of miR-200 family members in both tumor and serum samples were analyzed in this study. This systemic review and meta-analysis showed that elevated cancer tissue expression of miR-200 was associated with longer survival in patients with multiple carcinoma types. In contrast, high levels of miR-200 in serum were associated with poor prognosis.

Recently, two meta-analyses on the prognostic value of miR-200 were published. Shi and Zhang [46] evaluated seven ovarian cancer studies and showed that high expression of miR-200c may predict improved survival (OS: HR = 0.34, 95% CI 0.20–0.58; PFS: HR = 0.64, 95% CI 0.50–0.82). However, this study focused on ovarian cancer and cannot be applied to other cancer types due to population heterogeneity and a small sample size. Wu et al. [47] found that miR-200c was not significantly correlated with either OS (HR = 1.41, 95% CI 0.95–2.10; *P* = 0.09) or PFS (HR = 1.12, 95% CI 0.68–1.84; *P* = 0.67) in various types of cancer. However, considering that some miRNAs have similar functions as their target genes, evaluating a set of miRNAs is preferable compared with a single miRNA to increase the prediction power. For example, Song et al. identified a signature of 17 miRNAs, which included the miR-200 family, in patients with gastric cancer [24]. This miRNA risk signature remained a strong predictor of survival (*P* = 0.015 and *P* = 0.006 for OS and PFS, resp.) in a multivariate analysis, compared with analysis of an individual miR-200 family member. This suggests that a panel of miRNAs is a better predictor of survival than is an individual miRNA. Therefore, we evaluated all five miR-200 family members instead of a single miRNA in this meta-analysis.

The results of this meta-analysis showed a pooled HR of 0.70 (95% CI 0.54–0.91), demonstrating that increased miR-200 family expression in cancer tissues is associated with a favorable outcome (*P* = 0.007). Furthermore, in a subgroup analysis based on tumor type, a statistically significant difference in OS was observed between brain and pancreatic cancer subgroups, with pooled HRs of 0.51 and 0.35, respectively. Subgroup analyses also showed that miR-141 and miR-200b were associated with favorable OS, with pooled HRs of 0.40 and 0.58, respectively. The miR-200 family has regulatory functions in diverse biological processes. Zhu et al. described miR-141 as a significant tumor suppressor in pancreatic cancer, as it interferes with the proliferative pathway mediated by Yes-associated protein-1 [11]. In addition, the miR-200 family inhibits EMT by regulating a number of target genes such as ZEB1 and ZEB2 [5]. MiR-200c strongly suppressed mammary duct formation from normal mammary stem cells and tumor formation from breast cancer stem cells in vivo by targeting B lymphoma Mo-MLV insertion region 1 homolog, a regulator of stem cell self-renewal [48]. In addition, downregulation of miR-200 family members has been associated with resistance to cytotoxic chemotherapeutic agents and EGFR inhibitors [16, 22, 31, 49, 50]. In addition, this may be mediated by two antiapoptotic factors, B-cell lymphoma 2 and X-linked inhibitor of apoptosis protein [51]. Taken together, the miR-200 family can affect cancer progression by regulating various cell signaling and genetic pathways.

Interestingly, the miR-200 levels in plasma and tumor tissues had opposing associations with survival in this study. The pooled outcome from the OS and PFS analyses revealed HRs of 1.68 (*P* = 0.007) and 2.62 (*P* < 0.001), respectively, showing that increased circulating miR-200 family expression is associated with unfavorable survival. Similarly, Wu et al.'s meta-analysis indicated that higher blood levels of miR-200c were significantly associated with poor OS (HR = 2.10, 95% CI 1.52–2.90, *P* < 0.00001), but there was no significant association in tumor tissue (HR = 1.41, 95% CI 0.95–2.10; *P* = 0.09) [47]. MiR-200 family members are increased in the blood of patients with breast [34], prostate [35], esophageal [37], gastric [40], ovarian [52], and metastatic colorectal cancers [41]. MiR-200 expression is correlated with metastasis and relapse in breast cancer [34]. Moreover, expression of miRNA, including miR-200, may be an early predictor of chemotherapy outcomes in prostate and esophageal cancers [35, 37]. In 258 cases of colorectal cancer [41], high levels of plasma miR-141 were associated with unfavorable OS (HR = 2.40, 95% CI 1.182–4.86). The reason for the discrepancies between cancer tissue and circulating levels is likely explained by the different functions of miRNAs in extracellular vesicles compared with tissue miRNAs. Le et al. reported that miR-200 family members are secreted by highly metastatic epithelial breast cancer cells and that the secretion of these miRNAs results in increased metastatic potential in xenograft models [45]. The authors proposed that the miR-200 family is potentially involved in promoting the last step of the metastatic cascade in the development of macroscopic metastatic masses at distant sites.

It is unknown whether miRNA expression in the systemic circulation reflects their expression in cancer tissues. Some studies have shown no correlation between miR-200 levels in serum and tumor tissues [53]. However, Tsujiura et al. found that the levels of plasma oncomiRNAs, including miR-21 and miR-106b, may reflect tumor miRNA levels [54]. Furthermore, a previous meta-analysis of miR-21 demonstrated that high miR-21 expression in both tissues and the circulation predicted poor outcomes [55]. Clinically, circulating biomarkers have numerous advantages, including easy access for monitoring, and their evaluation is therefore preferred for predicting early diagnosis, prognosis, and individualized treatments. However, there are still many barriers to overcome before utilizing circulating miRNAs as diagnostic or prognostic biomarkers in the clinic. These barriers include clarifying miRNA correlations between tumor tissues and circulation, normalizing data from different studies using reference genes [56] or internal controls [57], and developing sensitive, specific, reliable, reproducible, and inexpensive detection methods. In addition, circulating miRNA expression can be significantly altered by physiological or pathological conditions, such as pregnancy, heart failure, or sepsis [57]. Therefore, further clarification on the clinical roles of circulating miR-200 family members in well-designed prospective studies is needed.

Our meta-analysis has several limitations. Marked heterogeneity among the subjects was present in the OS and PFS groups. The heterogeneity of the population was likely due to differences in sample size, baseline patient characteristics (e.g., age, cancer type, tumor stage, and treatment type), follow-up duration, detection methods, and cut-off values. Thus, we only selected high-quality studies using a quality assessment based on the Newcastle-Ottawa Scale. When the studies were stratified by tumor type, heterogeneity was no longer detected in the brain tumor and pancreatic cancer subgroups (*P* = 0.71 and *P* = 0.26, resp.).

In conclusion, our meta-analysis suggests that the miR-200 family members are potential biomarkers and accurate prognostic predictors in patients with various carcinomas. The decreased tumor expression of the miR-200 family was significantly associated with poor survival in patients with brain, pancreas, and ovarian cancers. In contrast, low circulating miR-200 levels were associated with a positive prognosis in patients with colon and breast cancers. For future clinical application, large prospective studies are needed to validate the prognostic values of circulating miR-200 in individual cancer types.

## Supplementary Material

Supplementary Table 1: Searching keywords combination according to searching engine.
Supplementary Figure 1: Forest plot of hazard ratios for the prediction of overall (A) and progression-free survival (B) by high-expressing tissue miR-200 family members according to tumor type.
Supplementary Figure 2: Forest plot of hazard ratios for the prediction of overall (A) and progression-free survival (B) by high-expressing serum miR-200 family members according to tumor type.
Supplementary Figure 3: Forest plot of hazard ratios for the prediction of overall (A) and progression-free survival (B) by high-expressing miR-200 family members according to individual tissue miRNA levels.
Supplementary Figure 4: Forest plot of hazard ratios for the prediction of overall (A) and progression-free survival (B) by high-expressing miR-200 family members according to individual serum miRNA levels.

## Figures and Tables

**Table 1:** Characteristics of the eligible studies evaluating high miRNA expression levels in tissue samples and patient survival data.

Study (year) (ref)	Country	Cancer	Stage	Test	Cut-off value	miRNA	Sample size	MFD	Newcastle-Ottawa Quality Assessment Scale		
									Selection	Compatibility	Outcome
Feng et al. (2015) [13]	China	HCC	NA	qRT-PCR/ISH	ROC	200a	115	120 m	★★	★	★★
Li et al. (2015) [17]	China	HCC	I–IV	qRT-PCR/ISH	NA	429	161	96 m	★★★★	★	★★★
Lu et al. (2015) [8]	China	GC	I–IV	qRT-PCR	Median	141	141	60 m	★★★	★	★★
Wang et al. (2015) [14]	China	Glioma	I–IV	qRT-PCR	NA	200b	123	5 y	★★★	★★	★★
Yao et al. (2015) [9]	China	BC	I–III	qRT-PCR	Comparison w/normal group	200b	278	10 y	★★★★	★	★★
Zhao et al. (2015) [18]^†^	China	NSCLC	II_B_-III_B_	qRT-PCR	Median	200c	78	40 m	★★★	★	★★
Cao et al. (2014) [19]	China	OC	I–IV	qRT-PCR	Median	200a/b/c	100	56 m	★★★★	★	★★★
Diaz et al. (2014) [20]	Spain	CRC	II	qRT-PCR	Maxstat R package	429	127	120 m	★★★	★★	★★
Kim et al. (2014) [21]	Korea	NSCLC	I–IV	qRT-PCR	Median	200c	72	125 m	★★★	★★	★★★
Li et al. (2014) [22]^†^	China	NSCLC	IIIB-IV	qRT-PCR	Minimum *P* value	200c	150	18.5 [**9.6**] m	★★★	★★	★★★
Liu et al. (2014) [23]	China	HCC	I–IV	ISH	Median	141	212	100 m	★★★	★	★★
Song et al. (2014) [24]^*∗*^	China	GC	I–IV	qRT-PCR	Median/Lowest quintile values	200a/b/c	373	112 m	★★★	★	★★
Tejero et al. (2014) [25]	Spain	NSCLC	I–III	qRT-PCR	Maxstat R package	141/200c	155	160 m	★★★	★★	★★★
Zhang et al. (2014) [26]^*∗*^	China	AST	III-IV	qRT-PCR	Median	200b	122	120 m	★★★★	★★	★★★
Zhu et al. (2014) [11]	China	PC	I–IV	qRT-PCR	Mean	141	94	200 m	★★★★	★	★★
Zhu et al. (2014) [15]	China	NSCLC	I–IV	qRT-PCR	ROC	429	70	30 m	★★	★	★★
Berghmans et al. (2013) [16]	Europe	NSCLC	IV (79%)	qRT-PCR	predicted score	200c	38	60 m	★★★	★★	★★
Huang et al. (2013) [27]	China	HCC	I-II	qRT-PCR	Mean	429	138	140 m	★★★	★	★★★
Li et al. (2013) [28]	China	CRC	I–III	qRT-PCR	Median	429	107	82 m	★★★★	★	★★★
Tang et al. (2013) [29]	China	GC	I–IV	ISH	Median	200b/c	126	NA	★★★	★	★★
Torres et al. (2013) [10]^†^	Europe	EEC	I–IV	qRT-PCR	Median/ROC	200c/429 [**429**]	30	150 m	★★★★	★	★★
Xiao et al. (2013) [30]	China	HCC	I–III	qRT-PCR	Mean	200a	120	60 m	★★★	★	★★★
Zhao at al. (2013) [12]	China	PC	I–IV	qRT-PCR	Median	141	40	50 m	★★	★	★★
Leskelä et al. (2011) [31]^*∗*^	Spain	OC	I–IV	qRT-PCR	Median	429 [**141**,** 200a/b/c**,** 429**]	72	128 m	★★	★	★★
Marchini et al. (2011) [32]^*∗*^	Italy	OC	I	qRT-PCR	Contal & O'Quigley method	200b/c (A)	89	240 m	★★	★	★★
						200c (B)	55				
Yu et al. (2010) [33]	Japan	PC	I–IV	qRT-PCR	Median	200c	99	101 m	★★★	★	★★★

[**Value**] indicates the microRNA type or maximum follow-up duration for progression-free survival.

MFD: maximal follow-up duration, AST: astrocytoma, BC: breast cancer, CRC: colorectal cancer, EEC: endometrioid endometrial carcinoma, EOC: epithelial ovarian cancer, ESC: esophageal squamous cancer, GC: gastric cancer, HCC: hepatocellular carcinoma, OC: ovarian cancer, PC: pancreatic cancer, PrC: castration-resistant prostate cancer, NSCLC: non-small-cell lung cancer, ROC, receiver operating characteristic analysis, NA: not available, mo: months, wk: weeks, and y: years.

^*∗*^Study reporting both overall survival and progression-free survival data.

^†^Study reporting only progression-free survival data.

**Table 2:** Characteristics of the eligible studies evaluating high miRNA expression levels in serum samples and patient survival data.

Study (year)	Country	Cancer	Stage	Test	Cut-off value	miRNA	Sample size	MFD	Newcastle-Ottawa Quality Assessment Scale		
									Selection	Compatibility	Outcome
Antolín et al. (2015) [34]^*∗*^	Spain	BC	I–IV	qRT-PCR	ROC	200c/141	57	265 [**235**] w	★★★	★★	★★★
Lin et al. (2014) [35]	Australia	PrC	IV	qRT-PCR	Median	200b	97	62 m	★★★	★	★★★
Liu et al. (2014) [36]	China	HCC	I–IV	qRT-PCR	Median	200a	136	50 m	★★	★	★★
Zhu et al. (2014) [15]	China	NSCLC	I–IV	qRT-PCR	ROC	429	70	30 m	★★	★	★★
Yu et al. (2013) [37]	China	ESC	III-IV	qRT-PCR	Median	200c	157	50 m	★★	★★	★★
Tanaka et al. (2013) [38]^*∗*^	Japan	ESC	I–IV	qRT-PCR	Comparison w/normal group	200c	64	2 y	★★★	★	★★
Toiyama et al. (2014) [39]	Japan	CRC	I–IV	qRT-PCR	ROC	200c	321	60 m	★★★	★	★★
Valladares-Ayerbes et al. (2012) [40]^*∗*^	Spain	GC	I–IV	qRT-PCR	MeanComparison w/normal group	200c	52	60 m	★★★	★★	★★★
Cheng et al. (2011) [41]	China	CRC	I–IV	qRT-PCR	ROC	141 (Tianjin)	156/102	50/100 m	★★★	★	★★★
	USA					141 (TexGen)					

[**Value**] indicates microRNA type or maximum follow-up duration for progression-free survival.

MFD: maximal follow-up duration, AST: astrocytoma, BC: breast cancer, CRC: colorectal cancer, EEC: endometrioid endometrial carcinoma, EOC: epithelial ovarian cancer, ESC: esophageal squamous cancer, GC: gastric cancer, HCC: hepatocellular carcinoma, OC: ovarian cancer, PC: pancreatic cancer, PrC: castration-resistant prostate cancer, NSCLC: non-small-cell lung cancer, ROC, receiver operating characteristic analysis, NA: not available, mo: months, wk: weeks, and y: years.

^*∗*^Study reporting both overall survival and progression-free survival data.
