# Supplementary material for: Prognostic Role of the MicroRNA-200 Family in Various Carcinomas: A Systematic Review and Meta-Analysis
Source: Biomed Res Int. 2017 Feb 22;2017:1928021. doi: 10.1155/2017/1928021 (PMC5340960; doi:10.1155/2017/1928021)

Supplementary table 1. Searching keywords combination according to searching engine.

| **Searching keywords combination in CINAHL(n= 25 articles)** |
| --- |
| mir-141, mir-200, mir-429 or miRNA-141, miRNA-200, miRNA-429 or microRNA-141, microRNA-200, microRNA-429, prognostic or prognosis or survival or outcome or tumor or cancer or neoplasm |
| **Searching keywords combination in Embase(n= 351 articles)** |
| mir-141, mir-200, mir-429 or miRNA-141, miRNA-200, miRNA-429 or microRNA-141, microRNA-200, microRNA-429, prognostic or prognosis or survival or outcome or tumor or cancer or neoplasm |
| **Searching keywords combination in Google Scholar(n=377 articles)** |
| allintitle: prognostic OR prognosis OR survival OR outcome OR tumor OR cancer OR neoplasm "mir 141"  allintitle: prognostic OR prognosis OR survival OR outcome OR tumor OR cancer OR neoplasm "miRNA 141"  allintitle: prognostic OR prognosis OR survival OR outcome OR tumor OR cancer OR neoplasm "microRNA 141"  allintitle: prognostic OR prognosis OR survival OR outcome OR tumor OR cancer OR neoplasm "mir 200"  allintitle: prognostic OR prognosis OR survival OR outcome OR tumor OR cancer OR neoplasm "miRNA 200"  allintitle: prognostic OR prognosis OR survival OR outcome OR tumor OR cancer OR neoplasm "microRNA 200"  allintitle: prognostic OR prognosis OR survival OR outcome OR tumor OR cancer OR neoplasm "mir 200a"  allintitle: prognostic OR prognosis OR survival OR outcome OR tumor OR cancer OR neoplasm "miRNA 200a"  allintitle: prognostic OR prognosis OR survival OR outcome OR tumor OR cancer OR neoplasm "microRNA 200a"  allintitle: prognostic OR prognosis OR survival OR outcome OR tumor OR cancer OR neoplasm "mir 200b"  allintitle: prognostic OR prognosis OR survival OR outcome OR tumor OR cancer OR neoplasm "miRNA 200b"  allintitle: prognostic OR prognosis OR survival OR outcome OR tumor OR cancer OR neoplasm "microRNA 200b"  allintitle: prognostic OR prognosis OR survival OR outcome OR tumor OR cancer OR neoplasm "mir 200c"  allintitle: prognostic OR prognosis OR survival OR outcome OR tumor OR cancer OR neoplasm "miRNA 200c"  allintitle: prognostic OR prognosis OR survival OR outcome OR tumor OR cancer OR neoplasm "microRNA 200c"  allintitle: prognostic OR prognosis OR survival OR outcome OR tumor OR cancer OR neoplasm "mir 429"  allintitle: prognostic OR prognosis OR survival OR outcome OR tumor OR cancer OR neoplasm "miRNA 429"  allintitle: prognostic OR prognosis OR survival OR outcome OR tumor OR cancer OR neoplasm "microRNA 429" |
| **Searching medical subject headings (MeSH) vocabulary in Pubmed(n= 440 articles)** |
| "MIRN141 microRNA, human" [Supplementary Concept] or "MIRN429 microRNA, human" [Supplementary Concept] or "MIRN200 microRNA, human" [Supplementary Concept] |

Supplementary Figure 1. Forest plot of hazard ratios for the prediction of overall (A) and progression-free survival (B) by high-expressing tissue miR-200 family members according to tumor type.

(A)


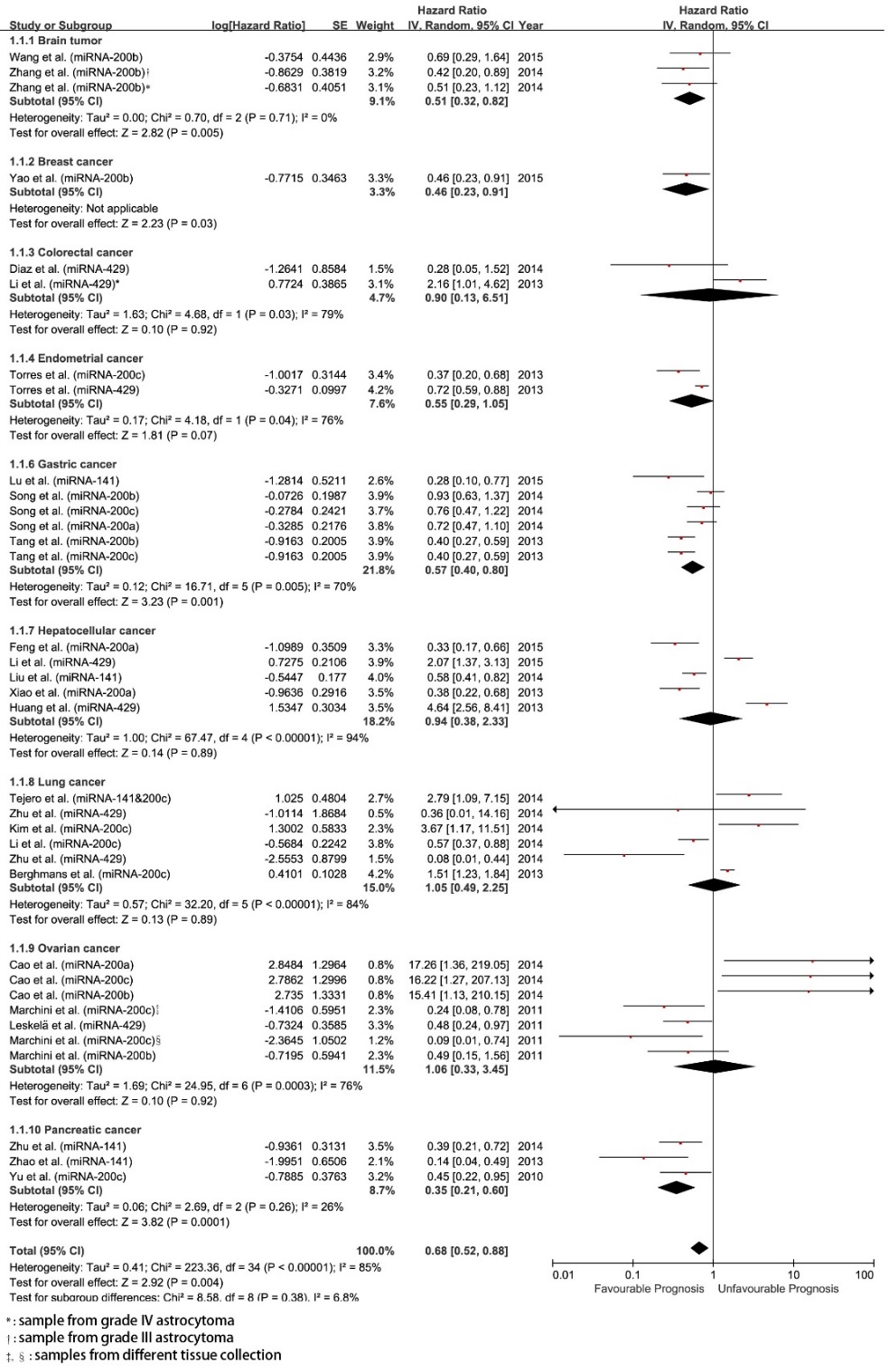


(B)


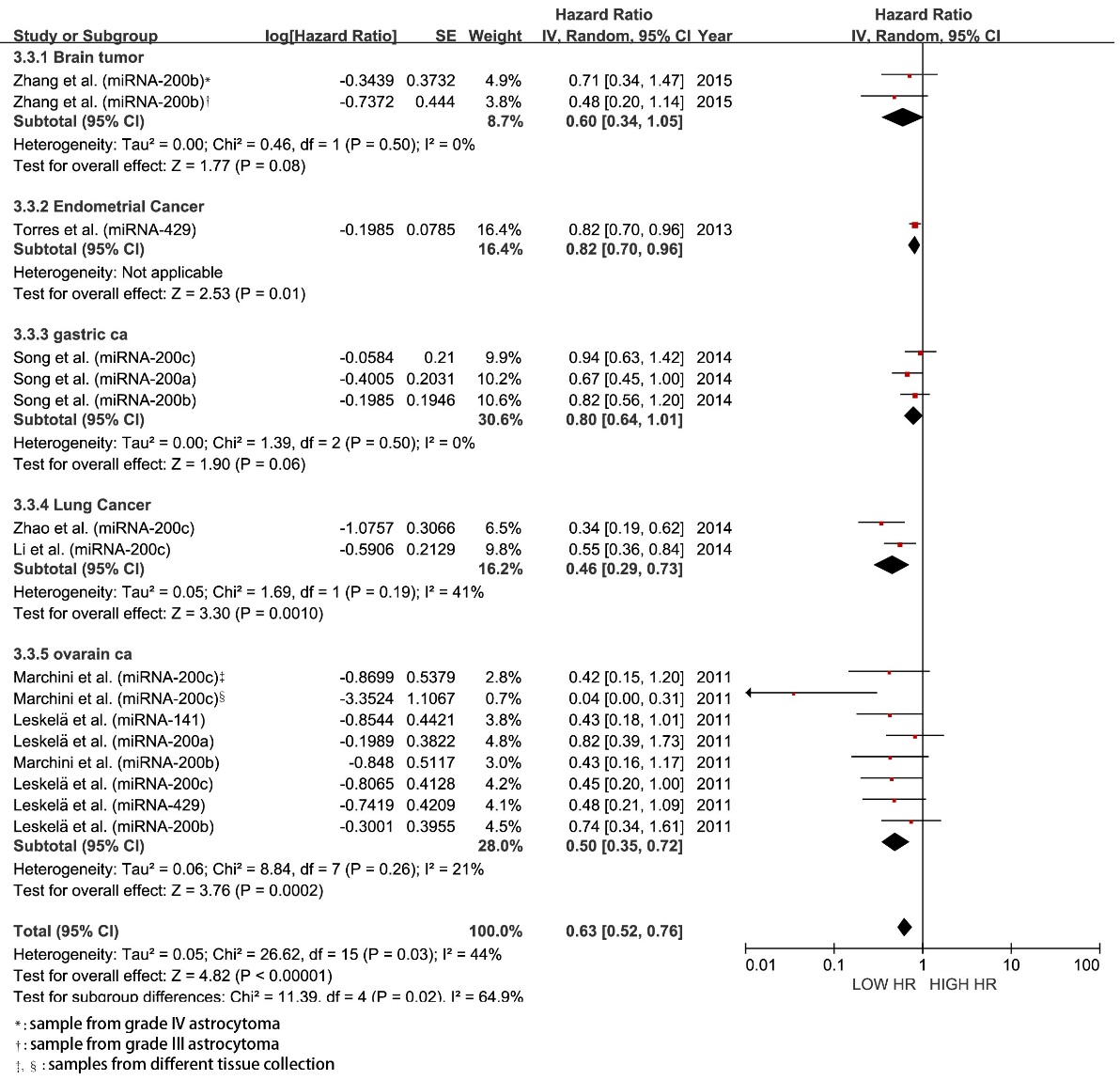


Supplementary Figure 2. Forest plot of hazard ratios for the prediction of overall (A) and progression-free survival (B) by high-expressing serum miR-200 family members according to tumor type.

(A)


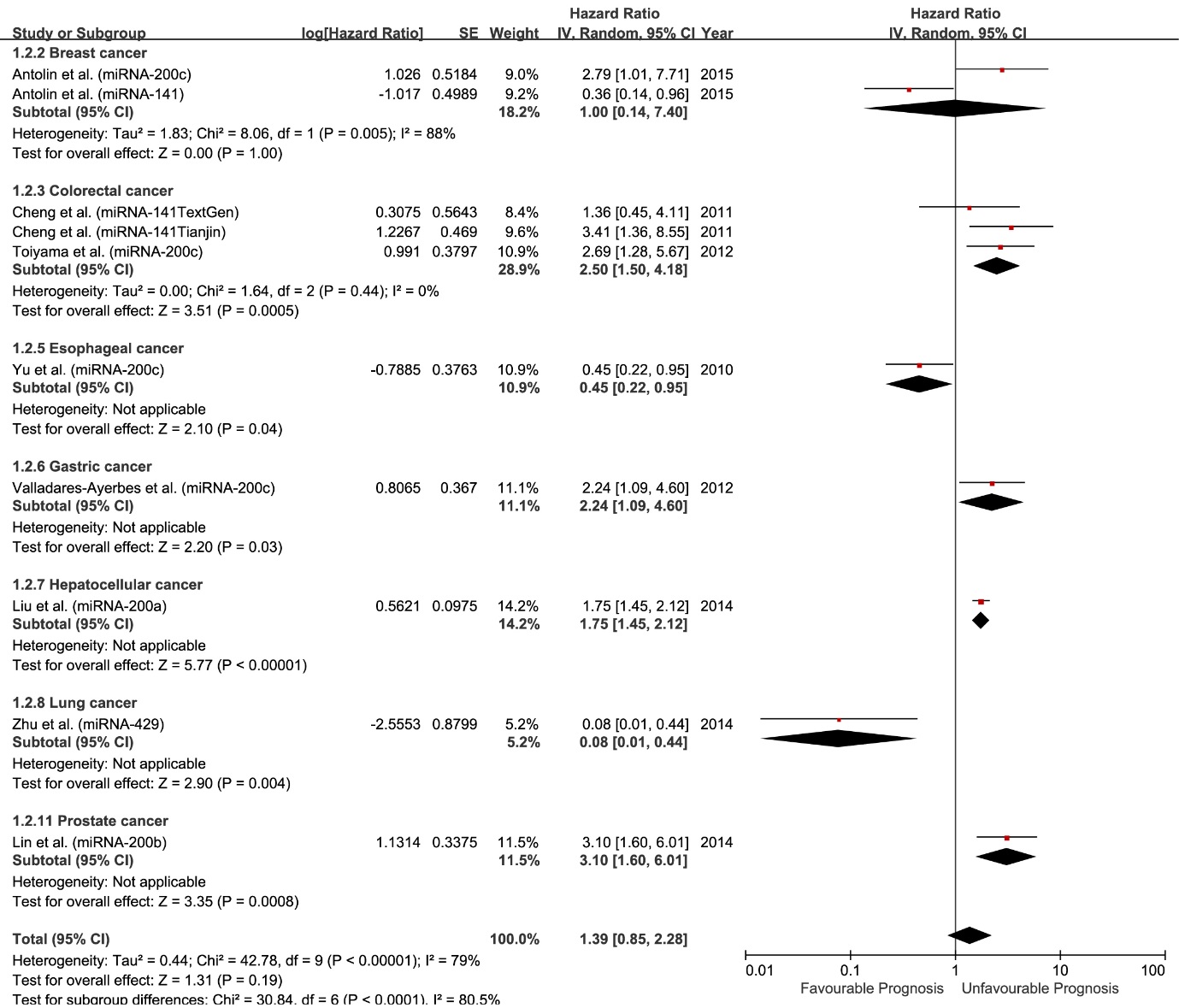


(B)


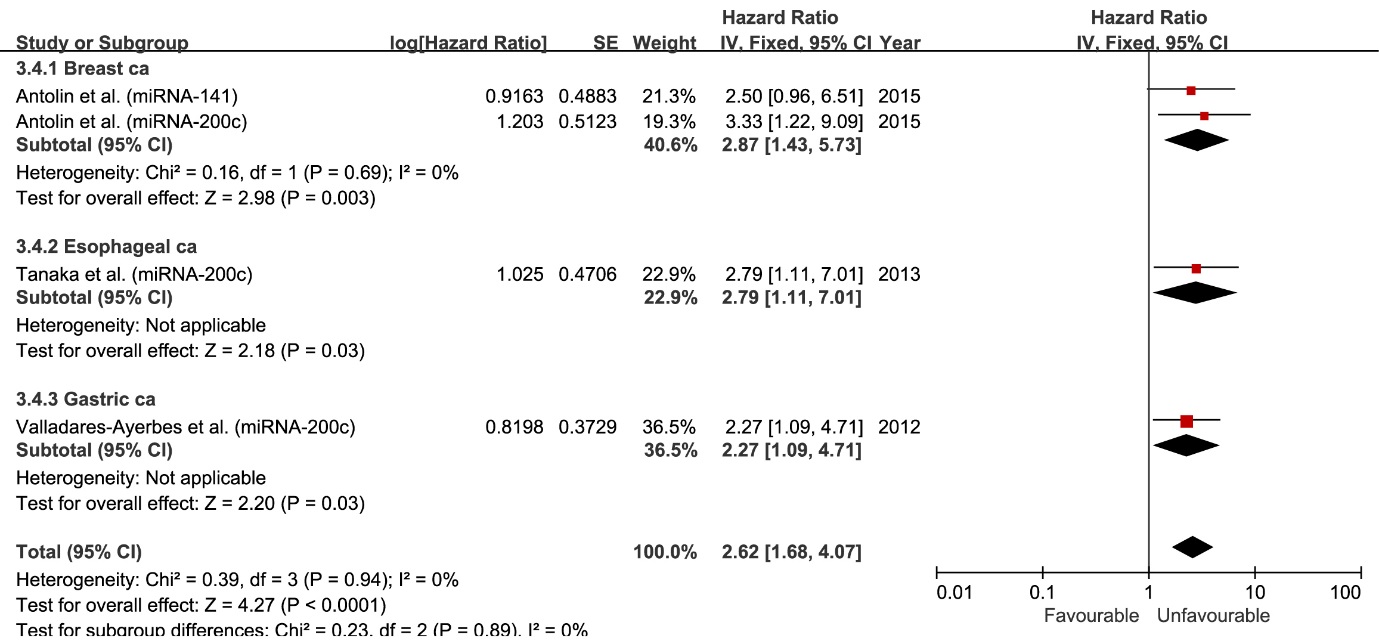


Supplementary Figure 3. Forest plot of hazard ratios for the prediction of overall (A) and progression-free survival (B) by high-expressing miR-200 family members according to individual tissue miRNA levels.

(A)


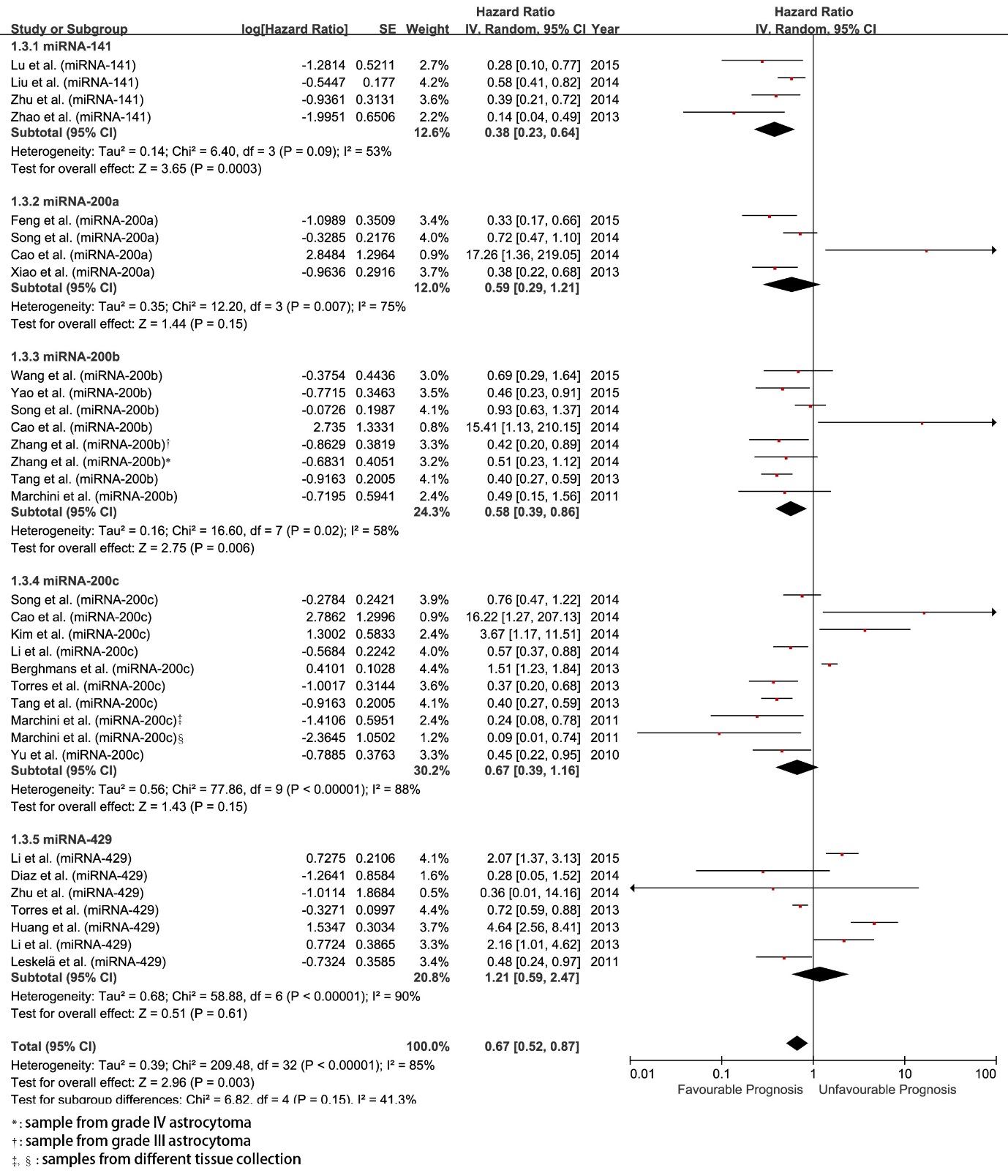


(B)


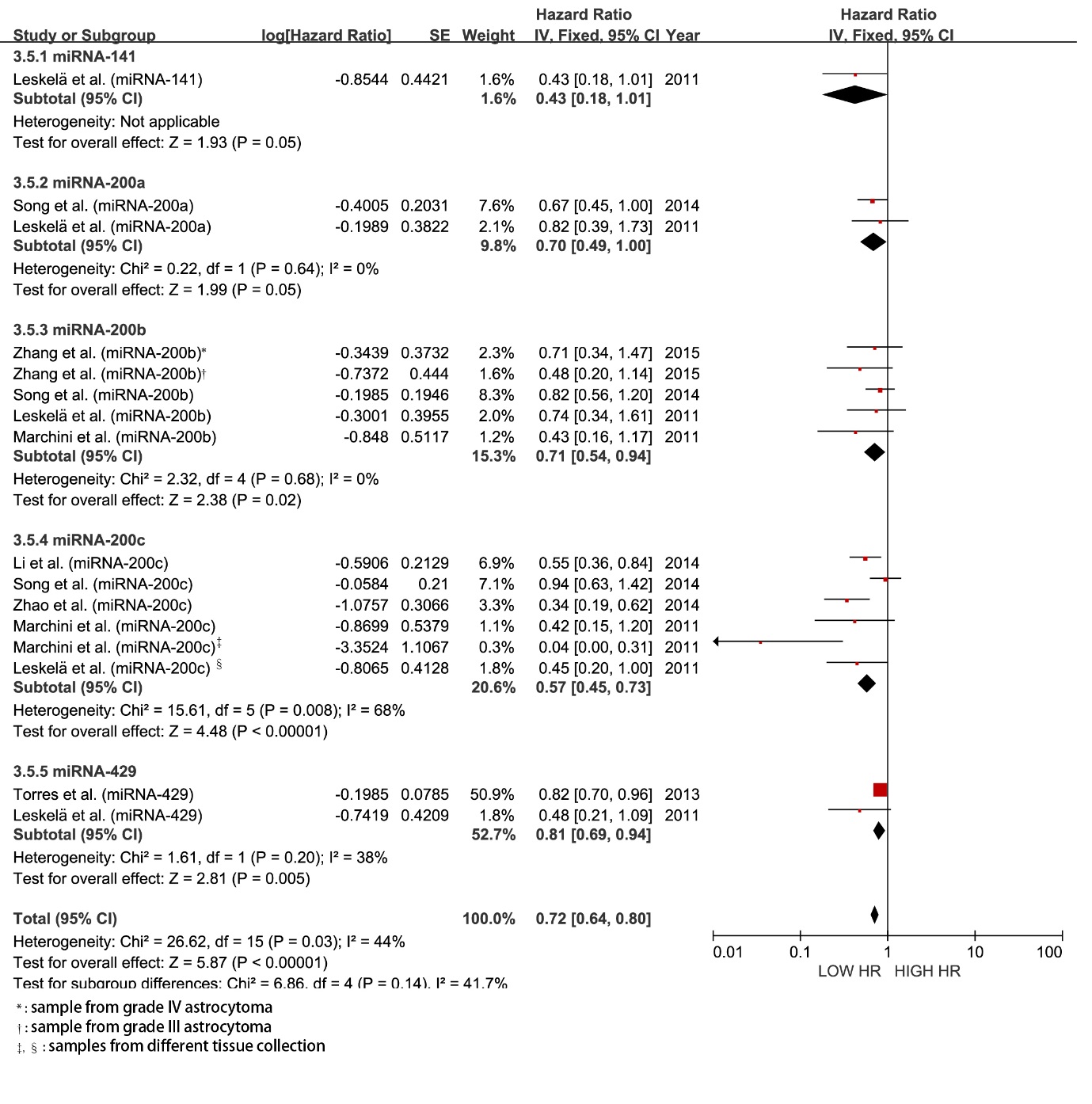


Supplementary Figure 4. Forest plot of hazard ratios for the prediction of overall (A) and progression-free survival (B) by high-expressing miR-200 family members according to individual serum miRNA levels.

(A)


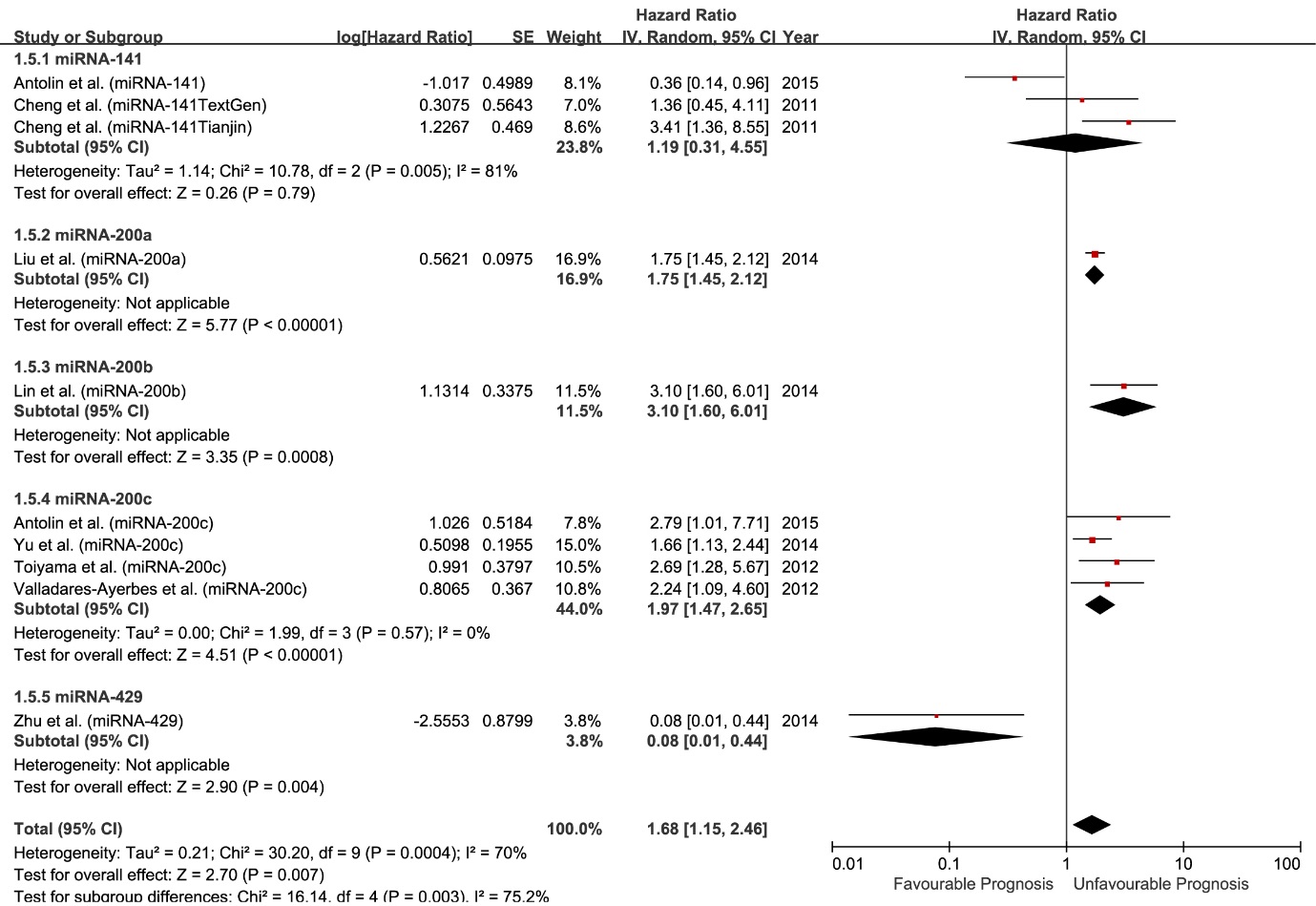


(B)


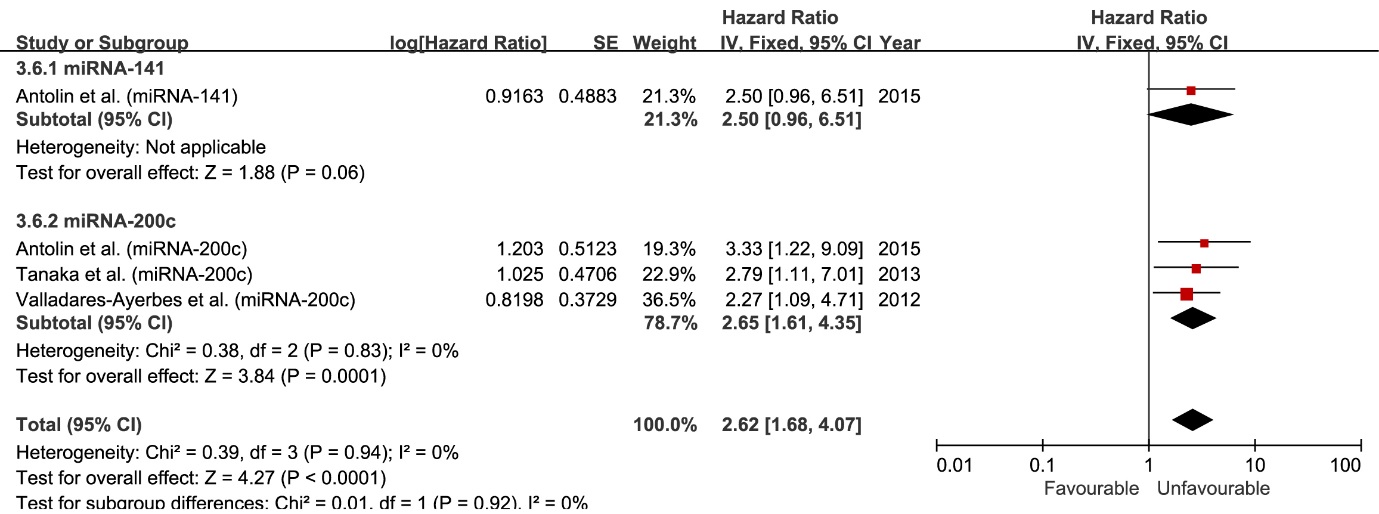

Supplement: Supplementary file 1 — Supplementary Table 1: Searching keywords combination according to searching engine. Supplementary Figure 1: Forest plot of hazard ratios for the prediction of overall (A) and progression-free survival (B) by high-expressing tissue miR-200 family members according to tumor type. Supplementary Figure 2: Forest plot of hazard ratios for the prediction of overall (A) and progression-free survival (B) by high-expressing serum miR-200 family members according to tumor type. Supplementary Figure 3: Forest plot of hazard ratios for the prediction of overall (A) and progression-free survival (B) by high-expressing miR-200 family members according to individual tissue miRNA levels. Supplementary Figure 4: Forest plot of hazard ratios for the prediction of overall (A) and progression-free survival (B) by high-expressing miR-200 family members according to individual serum miRNA levels. [file 1928021.f1.docx]
